# Glycerin/NaOH Aqueous Solution as a Green Solvent System for Dissolution of Cellulose

**DOI:** 10.3390/polym12081735

**Published:** 2020-08-03

**Authors:** Ke Li, Huiyu Yang, Lang Jiang, Xin Liu, Peng Lang, Bo Deng, Na Li, Weilin Xu

**Affiliations:** 1 State Key Laboratory of New Textile Materials and Advanced Processing Technologies, Wuhan Textile University, Wuhan 430200, China; dabing_ke@163.com (K.L.); hy-yang_wtu@hotmail.com (H.Y.); 15629129772@163.com (L.J.); xinliu_wtu@163.com (X.L.); 2 College of Material Science and Engineering, Wuhan Institute of Technology, Wuhan 430073, China; 3 College of Materials Science and Engineering, Qingdao University, Qingdao 266071, China; 17852156975@163.com; 4 School of Environmental Engineering and Chemistry, Luoyang Institute of Science and Technology, Luoyang 471023, China

**Keywords:** cellulose, glycerin, dissolution, green solvent, aqueous solution

## Abstract

Dissolving cellulose in water-based green solvent systems is highly desired for further industrial applications. The green solvent glycerin—which contains hydrogen-bonding acceptors—was used together with NaOH and water to dissolve cellulose. This mixed aqueous solution of NaOH and glycerin was employed as the new green solvent system for three celluloses with different degree of polymerization. FTIR (Fourier-transform infrared), XRD (X-ray diffractometer) and TGA (thermogravimetric analysis) were used to characterize the difference between cellulose before and after regenerated by HCl. A UbbeloHde viscometer was used to measure the molecule weight of three different kinds of cellulose with the polymerization degree of 550, 600 and 1120. This solvent system is useful to dissolve cellulose with averaged molecule weight up to 2.08 × 10^5^ g/mol.

## 1. Introduction

Cellulose has been put under the spotlight in the preparation of novel polymers and materials as one of the most affluent biopolymer sources in the world [1,2]. However, giant intra- and inter-molecule hydrogen bonds in the natural structure of cellulose result in its insolubility in both water and normal organic solvents, which greatly limits its application in industry [3].

Some solvent systems such as ammonium thiocyanate [4], calcium thiocyanate, sodium thiocyanate [5], lithium chloride/N, N-dimethylacetamide (LiCl/DMAc) [6,7] and NH_3_/NH_4_SCN [8], have been successfully applied to dissolve cellulose in the last century. However, resulting environmental pollution and high cost have confined these solvent systems as merely applicable at the lab scale.

Some green solvent systems including N-methylmorpholine-N-oxide (NMMO) [9], ionic liquid [10], water-based solvent systems [11] and mixed solvent systems—including amino acid ionic liquid/dimethyl sulfoxide (DMSO), tetra(n-butyl) ammonium hydroxide (TBAH) aqueous solution [12] and deep eutectic solvents (DESs) [13]—have been successively developed to dissolve cellulose. Although the mechanisms of cellulose dissolution varies with solvents, most researchers believe that—regardless of the molecule weight and crystallinity of cellulose [14]—the destruction of inter-molecule and intra-molecule hydrogen bonds in the complex structure of cellulose is a prerequisite to dissolve cellulose. Moreover, the interaction between the hydroxyl protons of D-dehydrated pyran glucose unit and dissociated solvent anions is the main driving force for cellulose dissolution [15].

Yuan et al. [16] first revealed that cellulose could be dissolved in a NaOH/urea aqueous solution after freezing the suspension into an ice-state, following a thawing process at room temperature under rigid agitation. This report opens the new window to dissolve cellulose in water-based solvent systems. To better understand the necessary of precooling procedure in the dissolving of cellulose, solid-state ^13^C-NMR [17], low temperature DSC [18], small-angle X-ray scattering [19,20] and synchrotron radiation micro-diffraction have been extensively applied. The results reveal that Na-cellulose complexes and hydrated alkali ions were two key factors that account mainly for the dissolving mechanism. The destruction of hydrogen bonds can be achieved by the formation of new hydrogen bonds between Na^+^ and hydroxyl groups of cellulose [21]. Moreover, the cellulose dissolution has been confined into an 8–9-wt% NaOH concentration region and became more remarkable at the temperature of or under four degrees Celsius [21].

Furthermore, Yuan et al. found that cellulose could be quickly dissolved in a precooled aqueous solution of LiOH/urea [18], NaOH/urea [16] or NaOH/thiourea [22] by generating a stable cellulose solution. In this, urea and thiourea acted as the hydrogen-bonding acceptor associated with the hydroxyl inside cellulose. These inter- and intra-chains associations stopped the regeneration of cellulose and ensures the solvation of cellulose.

Poly(ethylene glycol) (PEG)—another molecule which is possible candidate to stable the cellulose solution has also been successfully used by Yan to dissolve cellulose [23]. Instead, the oxygen atoms in the PEG chain are the hydrogen-bonding acceptor which stabilize the cellulose solution. The obtained cellulose solution in PEG/NaOH solvent system could be stable even for 30 days’ storage at room temperature at the cellulose concentration up to 13 wt%.

This is to say, any molecules with hydrogen-bonding acceptors are possible candidate for dissolving cellulose. Hydroxyl groups in a typical environmentally friendly molecule such as glycerin, could be alternative for the urea or thiourea to stable the cellulose solution [24].

In this study, a mixed aqueous solution of NaOH and glycerin was employed as a new green solvent system for three celluloses with different degree of polymerization (DP). Glycerin acts as a hydrogen bond acceptor which could prevent the ressociation of cellulose hydroxyl groups to form a stable and uniform solution. The proposed green solvent system could dissolve cellulose with a number average molecule weight of up to 2.08 × 10^5^ g/mol which was much higher than the reported 1.3 × 10^5^ g/mol [23].

## 2. Materials and Methods

### 2.1. Materials

Three kinds of celluloses (cotton linter pulps) with an α-cellulose content more than 95% were purchased from Shanghai Hengxin Chemical Reagent Co., Ltd (Shanghai, China). 

Their DPs were 550 (short fiber cotton linter pulps, referred to as SF-C), 600 (long fiber cotton linter pulps, referred to as LF-C), 1120 (high mechanical cotton linter pulps, referred to as HM-C), respectively. Corresponding physical properties of SF-C, LF-C and HM-C are shown in Table 1.

The cellulose was dried in vacuum at 35 °C overnight to remove the water content prior to use. Deionized water used in all solutions was taken from a Milli-Q Plus 185 water purification system (Millipore, Bedford, MA) and had a resistivity of 10–16 MΩ∙cm at 25 °C. Deuterium water (D_2_O, 99.9 atom% D) was purchased from Aladdin Reagent (Shanghai, China) Co., Ltd. Sodium hydroxide (NaOH), hydrochloric acid (HCl) and urea were purchased from Sinopharm Chemical Reagent Co., Ltd. (Shanghai, China).

### 2.2. Dissolution of Cellulose in Glycerin/NaOH Aqueous Solution

One gram of glycerin and 9.0 g of NaOH were added into 90 mL of deionized water to prepare the mixed aqueous solution of glycerin/NaOH. Then, 4.0 g of three kinds of cellulose were added into the mixture, respectively and allowed to swell for 6 h at room temperature. Then, the suspension was precooled down to -20 °C and held at that temperature overnight to make a solid frozen mass. The frozen solid was then stirred strongly under the action of a homogenizer (S10, Scientz company, Ningbo, China) at 20,000 rpm until the cellulose solution was completely thawed. Three kinds of homogeneous cellulose solutions were then finally obtained.

### 2.3. Preparation of Regenerated Cellulose

Around 2 mL 3-M HCl was added dropwise into 1 mL uniform cellulose solution and allowed to stand for 10 min. After the cellulose aggregates being completely collected by discarding the supernatant, the precipitate was washed three times by deionized water and then oven-dried at 40 °C for 20 h. Finally, pure and dry regenerated cellulose could be obtained.

### 2.4. Characterization

Photographs of dissolved and regenerated cellulose were taken with an Apple X mobile phone.

The microscopic morphology of cellulose before and after dissolution and further regenerated by 3-M hydrochloric acid (HCl) was analyzed with a scanning electron microscope (SEM, JSM-6510LV, JEOL Co., Ltd., Tokyo, Japan) using an accelerating voltage of 10 kV. The original cellulose and dried regenerated cellulose were cut into 5-mm × 5-mm samples and directly attached to the conductive adhesive for testing. Fifty microliters dissolved cellulose solution was directly dropped onto the surface of a 5 mm × 5 mm silicon wafer. The silicon wafer was cleaned with ethanol prior to solution deposition. Then, the silicon wafer was oven-dried at 45 °C for 1 hour and attached to a conductive adhesive on an aluminum sample holder for electron microscopy scanning.

^13^C-NMR spectrum of the cellulose solution was measured on a Bruker spectrometer (^13^C-NMR, AVANCE 400, BRUKER Co., Ltd., Karlsruhe, Germany). The number of scans was 1024; the time of each scan was 14.48 s. The solvent in this study was prepared by substituting D_2_O for H_2_O and the cellulose was dissolved to obtain a 4-wt% cellulose solution and 4 mL of this solution was measured for ^13^C-NMR spectrum in a nuclear magnetic tube with diameter of 5 mm and length of 20–25 cm.

An X-ray diffractometer (XRD, Bruke D8 Advance, Karlsruhe, Germany) was used to analyze the crystalline structure of the cellulose and the regenerated cellulose.

Thermogravimetric analysis (TGA) was carried out on a TA Instruments (TGA5500, New Castle DE, USA). A five-milligram sample was heated from 30 to 800 °C under nitrogen with 25-mL/min flow rate at a constant heating rate of 10 °C/min.

Fourier-transform infrared (FTIR, Nicolet NEXUS 670, Wisconsin, USA) spectroscopic analyses of all samples were done with a resolution of 2 cm^−1^ by averaging 64 scans in the range of 4000–400 cm^−1^. The FTIR spectra of dried original, dried regenerated cellulose and glycerin were taken under an attenuated total reflection (ATR) mode using corresponding accessory.

The viscosity of the cellulose in 4.6-wt% NaOH/ 15-wt% urea aqueous solution was measured at 25 ± 0.1 °C with a clean Ubbelohde viscometer (Ubbelohde viscometer, Youlaibo Technology Co., Ltd., Beijing, China). The used cellulose concentrations are 0.5 × 10^−3^ g/mL, 1.0 × 10^−3^ g/mL, 2.0 × 10^−3^ g/mL, respectively.

## 3. Result and Discussion

**Figure 1:**
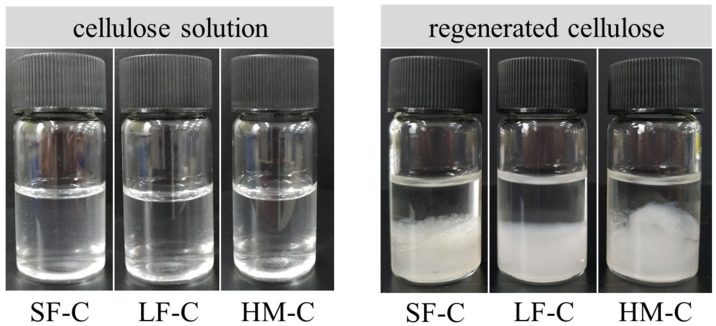
Photos of three kinds of cellulose aqueous solutions in 1.0-wt% glycerin/9.0-wt% NaOH and regenerated cellulose by 3-M HCl.

Figure 1 shows the photos of three kinds of cellulose aqueous solutions in 1.0-wt% glycerin/9.0-wt% NaOH before and after regeneration. Clear cellulose aqueous solutions without any aggregate in 1.0-wt% glycerin/9.0-wt% NaOH confirms that the good solubility of cellulose in glycerin/NaOH solvent system. After the addition of 3-M HCl, delamination appears by forming white precipitations at the bottom of solution which implies the regeneration of cellulose occurs.

**Figure 2:**
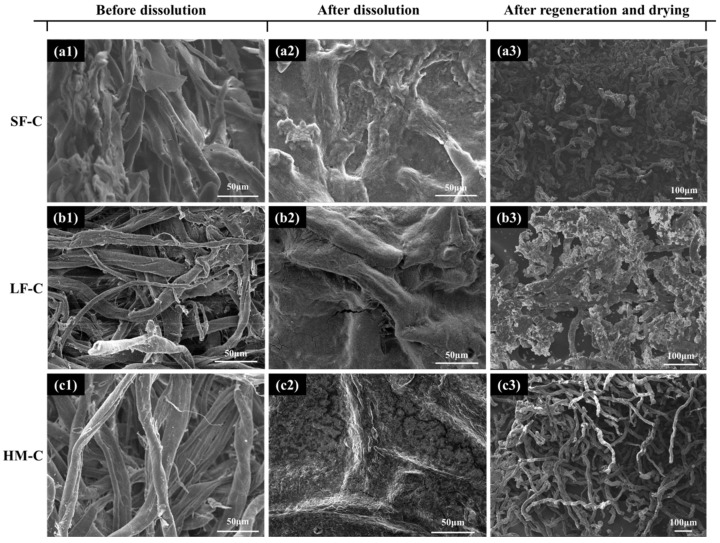
SEM images of (**a**) SF-C, (**b**) LF-P and (**c**) HM-C (a1, b1, c1) before dissolving the (a2, b2, c2) as-prepared cellulose solution under strong stir and (a3, b3, c3) the regenerated cellulose particles after the evaporation of water.

The dissolving status of cellulose in the new solvent system could be detected by SEM. As shown in Figure 2, SF-C, LF-C and HM-C show clear fiber-like morphology before dissolution. After completely dissolved in glycerin/NaOH solvent system, fiber-like morphologies disappeared, and a final homogeneous morphology could be obtained. This convinced the good dissolution ability of glycerin/NaOH solvent system to the cellulose. After further adding of HCl, the apparently strip morphology with much smaller size (compared with the cellulose before dissolution) may attribute to the reduced crystallinity which accompanied by increased amorphous area after cellulose regeneration [25].

**Figure 3:**
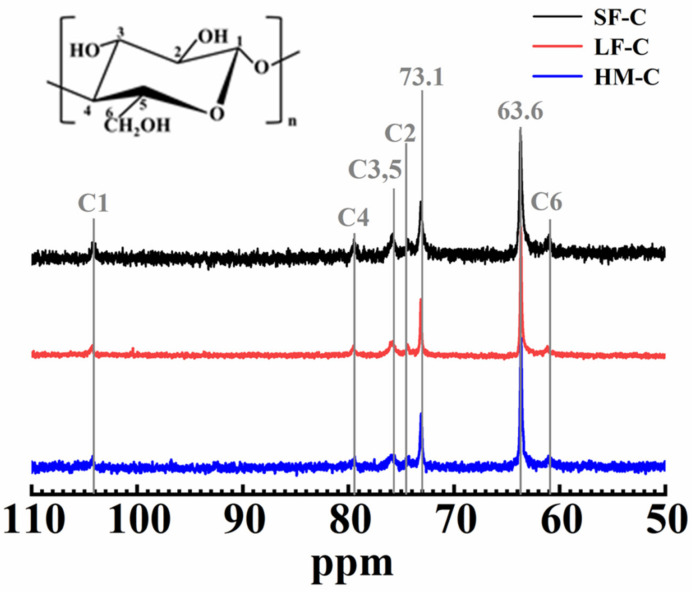
^13^C-NMR spectra of three kinds of 4.0-wt% cellulose in 1.0-wt% glycerin /9.0-wt% NaOH /D_2_O aqueous solution.

To further study the dissolution status of cellulose macromolecule in glycerin/NaOH solvent system. D_2_O was used to replace the deionized water to dissolve cellulose, SF-C, LF-C and HM-C in glycerin/NaOH solvent system. The concentration of cellulose was fixed to 4 wt% for three kind of celluloses. Figure 3 shows the ^13^C-NMR spectra of three different celluloses. Peaks located at 104.1 ppm, 79.5 ppm, 74.3 ppm and 61 ppm were ascribed to the carbon atom of C1, C4, C2, C6 as inserts in Figure 3. Twin peak located at 75.7 ppm was ascribed to the carbon atom of C3,5 [26]. Compared with the reported cellulose I obtained from the NaOH/urea solvent system, a higher magnetic field shifting from 79.2 ppm [27] to 79.5 ppm implies the destruction of intra-molecule hydrogen bonding, which is similar with that reported dissolution of wood pulp in LiCl/DMAc [28].

Peaks located at 73.1 ppm and 63.6 ppm were ascribed to the carbon in glycerin. Thus, we can conclude that three cellulose with different DPs could dissolve well in glycerin/NaOH. Moreover, the 1.0-wt% glycerin/9.0-wt% NaOH is the direct solvent of cellulose instead of a derivation aqueous solution.

**Figure 4:**
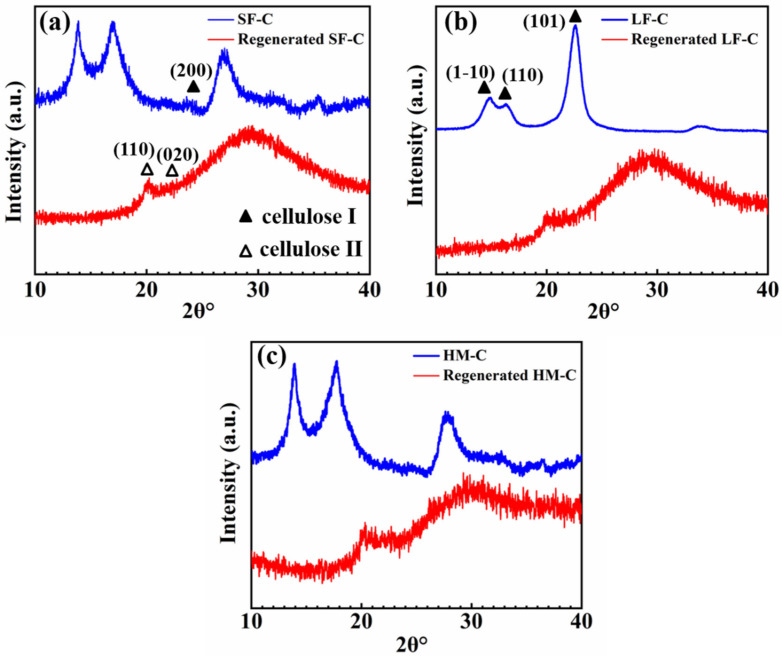
XRD patterns of the cellulose and regenerated cellulose from its glycerin/NaOH aqueous solution: (**a**) SF-C; (**b**) LF-C; (**c**) HM-C.

Figure 4 shows the XRD patterns of pristine and regenerated cellulose from glycerin/NaOH aqueous solution. Before regeneration, celluloses with different DPs showed characteristic 2θ peak at 14.9 ° and 16.4 ° which are correspond to (1–10), (110) lattice plane. Peak at 26.7° of SF-C and HM-C is in accordance with (200) lattice plane. However, the peak at 23.7° of LF-C is characteristic signal for (101) lattice plane. All these lattice planes are identical signals of cellulose I [29]. After regeneration, all celluloses showed peaks at 20.0 ° and 22.1 °, which are identical signals of (110) and (020) lattice planes of cellulose II, respectively [30]. These cellulose I to cellulose II transition convinced the successful regeneration of cellulose [31].

In addition, we calculated the crystallinity of celluloses with different DPs using Rietveld method [32]. The results show that the crystallinity of cellulose with different DPs in glycerol/NaOH aqueous solution changes significantly, and the destruction of molecular chain structure leads to a sharp decrease in crystallinity, as shown in Table 2. The sharply decreased crystallinity of cellulose after regeneration accounts mainly the excellent solubility of cellulose in NaOH/glycerin.

**Figure 5:**
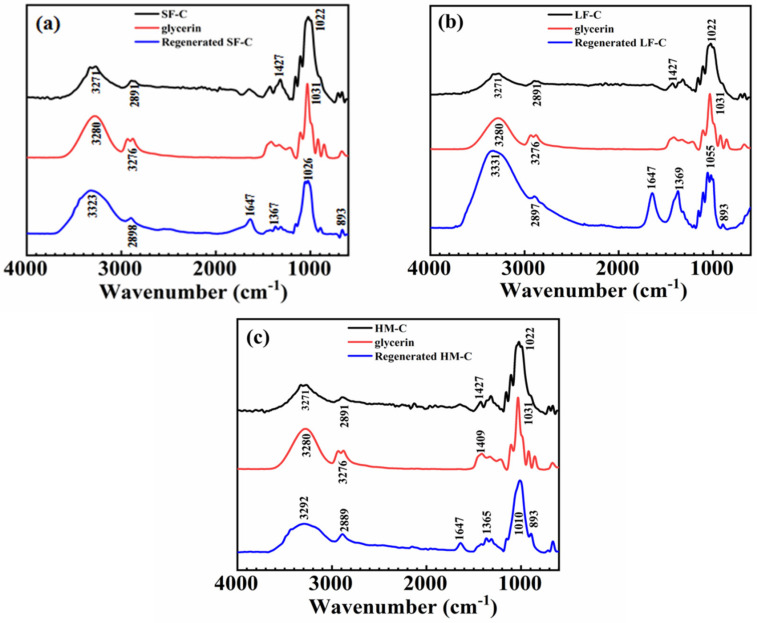
FTIR spectra of the cellulose, glycerin and regenerated cellulose from its glycerin/NaOH aqueous solution: (**a**) SF-C; (**b**) LF-C; (**c**) HM-C.

The regenerated cellulose could be obtained by adding diluted HCl into cellulose aqueous solution and followed by rinsing and drying. Figure 5 reveals the FTIR spectra of the cellulose, glycerin and regenerated for SF-C, LF-C and HM-C.

The bathochromic shift of hydroxyl from 3270 cm^−1^ to 3320 cm^−1^ after regeneration is due to the weakening effect of glycerin to the inter- and intra-molecule hydrogen bonding [33]. Peak around 2890 cm^−1^ is the stretching vibration of CH while that of 1427 cm^−1^ is the bending vibration of CH_2_ in pristine cellulose. The transition of CH_2_ around 1427 cm^−1^ (in pristine cellulose) to CH around 1369 cm^−1^ (in regenerated cellulose) after regenerating indicates the rotational isomer variation from C3–O3 and C6–O6. This further convinces the transition from cellulose Ⅰ to cellulose II [34].

The absorption peak around 893 cm^−1^ is the outward stretching vibration of asymmetric rings which corresponds to the vibration band of C5 and C6. The existence of this peak means the cellulose is well dissolved in the solvent [35].

Compared with spectrum of glycerin, peak at 1647 cm^−1^ in regenerated cellulose indicates the trace amount of glycerin residue [36].

**Figure 6:**
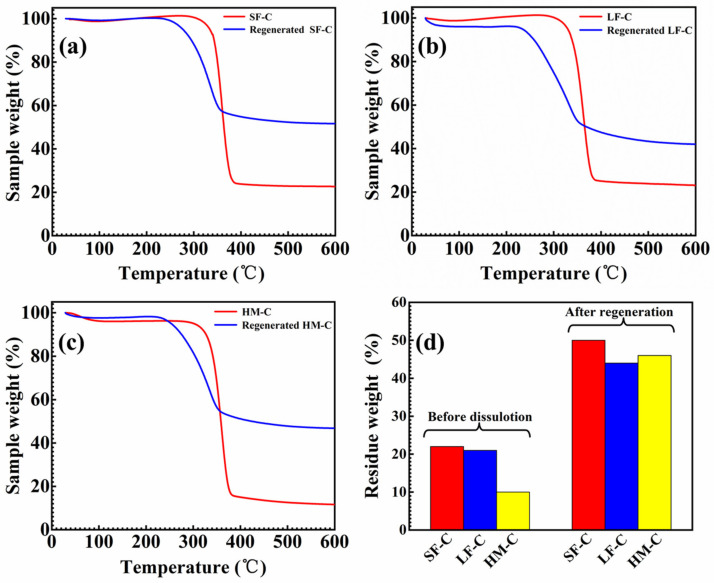
Thermal analysis of the cellulose and regenerated cellulose from its glycerin/NaOH aqueous solution: (**a**) SF-C; (**b**) LF-C; (**c**) HM-C; (**d**) comparison of TGA of different state cellulose.

Figure 6 shows the contrastive TGA curves of three kinds of cellulose and corresponding regenerated cellulose. The decomposition temperature of all pristine celluloses starts from 290 ℃ while that for regenerated cellulose shows a sharply decreased temperature of 230 ℃. This is because a large number of regular hydrogen bonds are destroyed during the regeneration in crystalline region which lowers the thermal stability of cellulose [37]. Char residual weight percentages of pristine and regenerated celluloses are summarized in Figure 6d. Much higher amount of char from regenerated cellulose is due to the existed bigger amorphous zone which is favorable for the forming of pyrolytic char [38].

**Figure 7:**
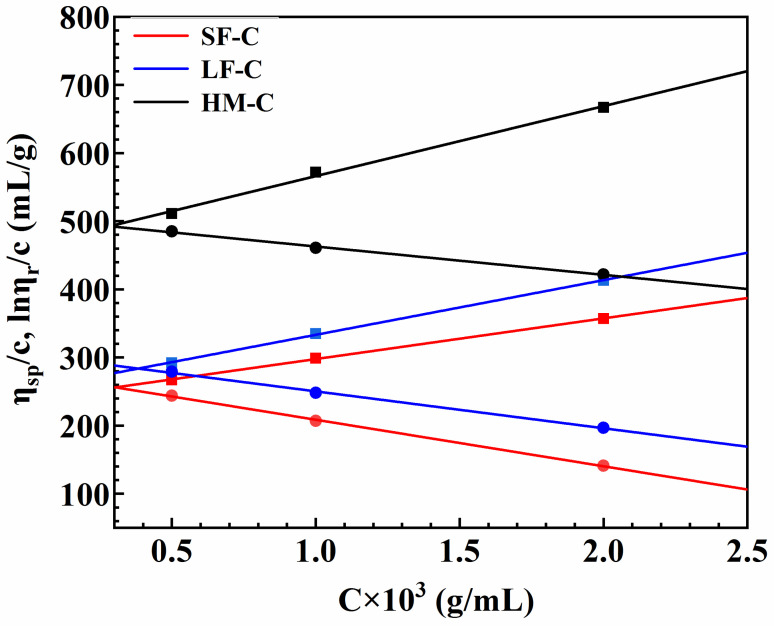
Dependence of intrinsic viscosity ([η]) on the concentration of three kinds of cellulose in 4.6-wt% NaOH/15-wt% urea aqueous solution at 25 °C.

The **d**ependence of intrinsic viscosity on the concentration of three kinds of cellulose in 4.6-wt% NaOH/15-wt% urea aqueous solution at 25℃ was plotted in Figure 7.

Clear intersection points in all Huggins–Kraemer curves demonstrate that the glycerin/NaOH system is good solvent for cellulose with different DPs. Intercepts which indicate the intrinsic viscosity for SF-C, LF-C and HM-C are 238 mL/g, 253 mL/g and 464 mL/g, respectively.

According to the formula (1), the calculated number averaged molecular weights of SF-C, LF-C and HM-C are 8.77 × 10^4^ g/mol, 9.49 × 10^4^ g/mol, 2.08 × 10^5^ g/mol, respectively. According to the literature [39], the K and α value used here are 3.72 × 10^–2^ cm^3^/g and 0.77, respectively.

(1) $$\left[\eta \right]={KM}^{\alpha }$$

All these results indicate the glycerin/NaOH is a novel good solvent system to dissolve cellulose with the molecular weight up to 2.08 × 10^5^ g/mol. Zhang et al. [22] reported that the NaOH/thiourea system can dissolve cellulose with a viscosity average molecular weight of 2.0 × 10^5^ while introduced thiourea will bring about secondary pollution.

## 4. Conclusions

In this communication, we developed a novel green solvent system, glycerin/NaOH, to dissolve cellulose. The aqueous solution of 1.0-wt% glycerin/9.0-wt% NaOH could dissolve the cellulose well and form a homogeneous solution. Glycerin acts as the hydrogen-bonding acceptor which could stop the reassociation of hydroxyl groups of cellulose to form homogeneous solution. Moreover, also this method is applicable to dissolve cellulose with the number averaged molecular weight up to 2.08 × 10^5^ g/mol.

## Figures and Tables

**Table 1:** Physical properties of cellulose with different degrees of polymerization (DPs).

	DP	α-Cellulose /%	Viscosity/mPa·s	Ash/%	Fe/ppm	Alkali Absorption Value/%	H_2_O/%	Whiteness/%	Small Dust/mm^2^/kg
SF-C	550	95.1	9.2	0.1	11	513	13.1	78	154
LF-C	600	96.0	11.4	0.07	8	699	13.2	80	79
HM-C	1120	98.6	29.9	0.06	11	582	8.7	82	88

**Table 2:** Crystallinity of cellulose with different DPs before and after regeneration.

Sample	Before Regeneration (%)	After Regeneration (%)
SF-C	47.3±1.7	1.6±0.2
LF-C	57.7±0.6	4.1±0.3
HM-C	61.6±1.7	14.9±0.9
